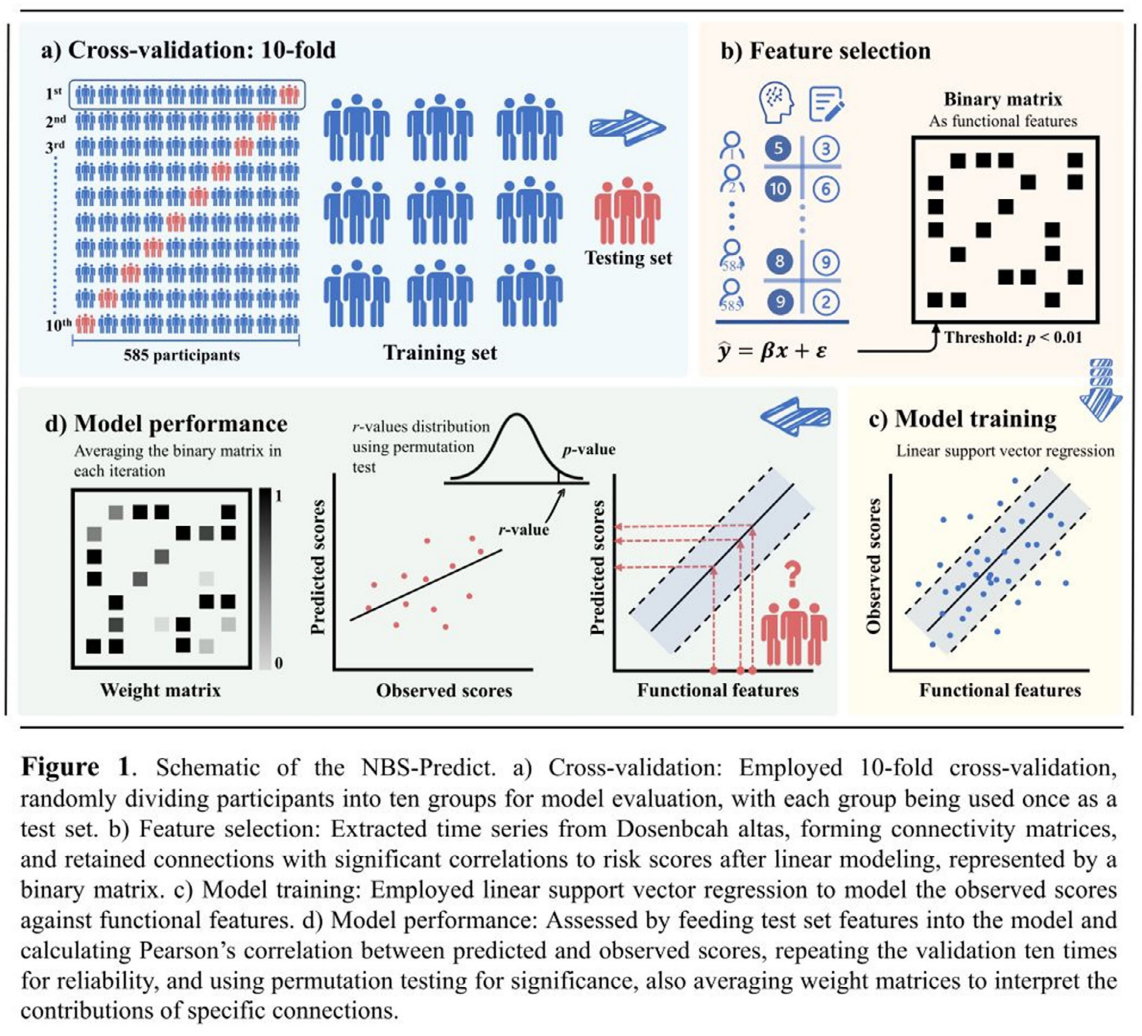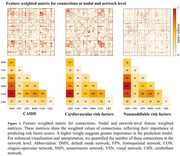# Brain‐based predictions of cardiovascular risk factors in midlife populations at risk of dementia

**DOI:** 10.1002/alz.086417

**Published:** 2025-01-09

**Authors:** Bolin Cao, Feng Deng, Qing Qi, Maria‐Eleni Dounavi, Graciela Muniz‐Terrera, Paresh Malhotra, Ivan Koychev, John T O'Brien, Craig Ritchie, Brian Lawlor, Lorina Naci

**Affiliations:** ^1^ Trinity College Dublin, Dublin Ireland; ^2^ Global Brain Health Institute, Trinity College Dublin, Dublin Ireland; ^3^ Trinity College Institute of Neuroscience, School of Psychology, Trinity College Dublin, Dublin Ireland; ^4^ Department of Psychiatry, University of Cambridge, Cambridge UK; ^5^ Edinburgh Dementia Prevention, University of Edinburgh, Edinburgh UK; ^6^ Department of Social Medicine, Ohio University, Athens, OH USA; ^7^ UK Dementia Research Institute Centre for Care Research and Technology, London UK; ^8^ Department of Brain Sciences, Imperial College London, London UK; ^9^ University of Oxford, Oxford UK; ^10^ Scottish Brain Sciences, Edinburgh UK

## Abstract

**Background:**

Dementia, particularly Alzheimer's disease (AD), is a significant public health concern, with midlife emerging as a critical period for preventive intervention (Livingston, 2017). Dementia's heterogeneity renders single risk factor insufficient for accurate identification of individuals at risk (Stephen, 2021). Multifactorial risk scores, such as the cardiovascular risk factors, aging, and dementia (CAIDE) score (Kivipelto, 2006), which include both cardiovascular (blood pressure, cholesterol, BMI, physical inactivity) and non‐modifiable factors (age, sex, APOE ε4 genotype), are vital in assessing dementia risk. This study, using the network‐based statistic (NBS)‐Predict model (Serin, 2021), aimed to explore the functional brain architecture associated with these risk factors, aiding in personalized prevention and intervention strategies for dementia.

**Method:**

Resting‐state fMRI data, CAIDE, cardiovascular and non‐modifiable risk scores, and lifetime of experiences questionnaire (LEQ) data were analyzed from 585 healthy participants (females/males=207/378, mean age=50.9) in the PREVENT‐Dementia study (Ritchie, 2023). Using the Dosenbach atlas, functional connectivity (FC) matrices were constructed post‐data preprocessing. The NBS‐Predict model, employing a linear support vector machine with 10‐fold cross‐validation, feature selection at p<0.05, and 1000 permutations, predicted CAIDE, cardiovascular, and non‐modifiable risk scores (Figure 1). A hierarchical regression model assessed the impact of midlife LEQ score on FC linked to cardiovascular risk factors, with LEQ scores, age, sex, education years, and mean framewise displacement as independent variables.

**Result:**

NBS‐Predict models significantly predicted the CAIDE (r=0.214, p<0.001), cardiovascular (r=0.201, p<0.001), and non‐modifiable (r=0.237, p<0.001) risk factors scores. Similar FC patterns were observed between the CAIDE and cardiovascular risk scores, particularly involving the somatomotor and cingulo‐opercular networks (Figure 2a), contrasting with distinct patterns for non‐modifiable risk factors. The non‐specific LEQ score positively correlated with FC in regions affected by cardiovascular risk factors (β=0.001, p=0.017).

**Conclusion:**

The study demonstrated a significant overlap in FC patterns between CAIDE and cardiovascular risk factors in midlife, distinct from patterns associated with non‐modifiable risk. This suggested different neurobiological pathways influencing dementia risk in midlife, emphasizing the importance of personalized dementia prevention strategies. The findings highlighted the positive impact of an active and engaged lifestyle on brain health, reinforcing the need for early intervention targeting cardiovascular health for dementia risk reduction.